# The role of BH3-only protein Bmf in the pathogenesis of dominant negative hepatocyte nuclear factor-1 –induced mature-onset diabetes of the young in transgenic mice

**DOI:** 10.1186/1753-6561-9-S7-A25

**Published:** 2015-10-27

**Authors:** Fahd Alkhalifah, Shona L Pfeiffer, Luise Halang, Heiko Dussman, Jochen H M Prehn

**Affiliations:** 1 School of Medicine, Royal College of Surgeons in Ireland, Dublin, Ireland; 2 Department of Physiology and Medical Physics, Royal College of Surgeons in Ireland, 123 St. Stephen's Green, Dublin 2, Ireland

## Introduction

Maturity Onset Diabetes of the Young 3 (MODY3) is the most common monogenic form of diabetes, characterized by early age of onset (before the age of 25), autosomal dominant transmission and severe defect in insulin secretion [1,2]. MODY accounts for 2-5% of Non-Insulin Dependent Diabetes Mellitus (NIDDM) cases, with MODY3 identified as the most common and severe form, accounting for 65% of all MODY cases and results from loss of function mutations of the transcription factor Hepatocyte Nuclear Factor-1 α (HNF1α). As a result of this, pancreatic islets show reduction in glucose-stimulated insulin secretion response and in beta cell mass, hallmarks of MODY3. Previous work in this laboratory has shown that induction of dominant negative mutant-HNF1a expression results in bioenergetic stress, activation of AMPK and induction of pro-apoptotic BH3-only family protein, Bmf [3].

## Methods

To study the role of Bmf (Bcl-2 modifying factor) in the pathogenesis of MODY3, immunohistochemical staining of male and female pancreatic islets for insulin- and glucagon-positive expression utilising confocal microscopy followed by Image J analysis were used to investigate the effect of *bmf* gene expression knockout on beta cell mass and islet organisation in a transgenic mouse model of MODY3.

## Results

*Bmf* gene expression knockout was observed to significantly increase beta cell mass in DN-HNF1a-expressing transgenic male pancreas (*p=0.04)* but had no observable effect on transgenic female pancreas. Surprisingly, islet disorganisation was seen in both male and female transgenic mice and was not rescued by *bmf* knockout.

## Conclusions

Data generated from this study indicate a possible role for Bmf in beta cell mass reduction and thereby the pathogenesis of MODY3 but demonstrates no effect on islet organisation. These data can be built upon by further research to examine in greater detail how Bmf contributes to the characteristic loss of beta cell mass in MODY3 and in mediating DNHNF1a-induced apoptosis.
